# Sense of coherence and quality of life in adolescents with heart disease

**DOI:** 10.1590/1984-0462/2022/40/2021104IN

**Published:** 2022-05-06

**Authors:** Virgínia Menezes Coutinho, Gabriele Lima de Araújo, Maria Carlla Aroucha Lyra, Aronita Rosenblatt, Mônica Vilela Heimer

**Affiliations:** a Universidade Federal de Pernambuco, Recife, PE, Brazil.; b Universidade de Pernambuco, Camaragibe, PE, Brazil.

**Keywords:** Adolescent, Sense of coherence, Quality of life, Heart diseases, Adolescente, Senso de coerência, Qualidade de vida, Cardiopatias

## Abstract

**Objective:**

The aim of this study was to assess the association between the Sense of Coherence (SoC) and Quality of Life (QoL) in adolescents with heart disease.

**Methods:**

A cross-sectional study was carried out with 164 adolescents with congenital or valvular heart disease, aged between 10 and 18 years, treated in a referral center in the city of Recife – Brazil. The information collected contains census data, type of heart disease, economic status identified according to the Brazilian Criteria for Economic Classification (ABEP), as well as an evaluation of the SoC and the QoL.

**Results:**

The SoC (50.09) and QoL (72.23) exhibited high average scores. The SoC was positively correlated with all dimensions of the QoL scale (p<0.001). The social and school dimensions, respectively, presented the highest and lowest scores. The linear regression analysis revealed that the SoC influenced the school and emotional dimensions.

**Conclusions:**

This study demonstrates that the SoC is a protective factor in the life of adolescents. This factor helps on the improvement of perception of QoL and on successfully dealing with daily adversities and chronic stress.

## Resumo

**Objetivo:**

Avaliar a associação entre Senso de Coerência (SOC) e Qualidade de Vida (QV) em adolescentes cardiopatas.

**Métodos:**

Foi realizado um estudo transversal com 164 adolescentes portadores de cardiopatias congênitas ou valvares, com idade entre 10 e 18 anos, atendidos em um Centro de Referência na cidade de Recife - Brasil. As informações coletadas contêm dados censitários, tipo de cardiopatia, situação econômica identificada de acordo com os Critérios Brasileiros de Classificação Econômica (ABEP), além de uma avaliação do Senso de Coerência e da Qualidade de Vida.

**Resultados:**

Tanto o Sentido de Coerência (50,09) quanto a Qualidade de Vida (72,23) exibiram valores médios elevados. Senso de Coerência foi positivamente correlacionado com todas as dimensões da Qualidade de Vida (p<0,001). As dimensões social e escolar foram as pontuações mais altas e mais baixas, respectivamente. A análise de regressão linear revelou que o Sentido de Coerência influenciou as dimensões escolar e emocional.

**Conclusões:**

Este estudo demonstra que o Sentido de Coerência é um fator de proteção na vida dos adolescentes. Esse fator auxilia na melhora da percepção de Qualidade de Vida e no enfrentamento com sucesso das adversidades do dia a dia e do estresse crônico.

## INTRODUCTION

According to the present Brazilian Ministry of Health, chronic and disabling diseases are often highly prevalent in both adolescents and young adults.^1^ Approximately 9.7–11% of adolescents aged between 12 and 19 years tend to have some types of chronic disease, namely, heart disease.

The ability of an individual to deal with the stress experienced during a certain disease is an important aspect to be investigated by health professionals, with the individual’s phenotype, family context, and life circumstances having to be considered.^2^ One of the aspects that has been studied when investigating the ability of facing stress is the Sense of Coherence (SoC), which is accounted as a personal attribute to protect an individual against the harmful consequences of adversity.^3,4^

The SoC is the key concept within the salutogenic theory proposed by Aaron Antonovsky.^4^ This theory is regarded as a new medical approach for assessing chronic health conditions in individuals or within specific groups.^5^ According to this theory, stressors are intrinsic to the human condition. Therefore, the higher the SoC, the better individuals can deal with these agents, consequently having a good understanding of their health and a better Quality of Life (QoL).^6–8^

The World Health Organization Quality of Life Group (WHOQOL)^9^ defined QoL as “an individual perception of one’s position in life, within the context of the culture and value systems in which they live in connection to their goals, expectations, standards and concerns.”

Since most of the health behaviors are developed in adolescence, it is of paramount importance to analyze this age group. In this regard, studying the SoC of an adolescent with heart disease provides a better understanding of how this age group reacts to the disease and its treatment, enabling to establish strategies to tackle the situation, thus improving their QoL.^10^ The present study aimed at evaluating the association between SoC and QoL, considering demographic and clinical data on adolescents with heart disease.

## METHOD

A cross-sectional study was carried out with 164 adolescents with heart disease, treated in a reference center for pediatric cardiology in the Brazilian city of Recife. Participants were selected according to the following criteria: aged between 10 and 18 years, with a diagnosis of congenital heart and/or valvar disease, according to a second medical diagnosis obtained from the patient’s medical record. Patients with mental disorders and those with motor and/or cognitive impairments were excluded from the study, which would have prevented the application of the instruments used.

The data collection was carried out through individual interviews and analysis of medical records of the participants. Initially, a sociodemographic questionnaire, previously prepared, was applied by interview. This questionnaire consisted of data such as age, gender, parent’s schooling, and approximate family income. The clinical characterization of the adolescents was obtained by looking at medical records, mainly the type of heart disease and associated symptoms.

The SoC was obtained in its short version, adapted and validated to the Brazilian population by Bonanato et al.^11^ In turn, the QoL assessment was carried out through the Peds QL 4.0 (Pediatric Quality of life Inventory^TM^) Instrument,^12,13^ with the economic classification being assessed according to the Economic Classification questionnaire rating of Brazil/2011 (ABEP).

The present study applied an adapted version by Bonanato et al.,^11^ which showed fewer unanswered questions and extreme responses, thus indicating a greater capacity to understand the issues analyzed. However, as this version was validated and adapted to mothers of preschoolers, a separate study was conducted with a sample of adolescents in order to test the reproducibility of this instrument. The instrument proved to be valid and consistent, with a Cronbach’s alpha of 0.74, and a weighted kappa ranging from 0.61 to 1.00.

The instrument consisted of 13 questions answered on a 5-point Likert scale, with intermediate responses and semantic limits for extreme responses located. The questions consisted of three fields (management, understanding, and meaning). However, according to Eriksson and Lindström,^14^ these fields cannot be analyzed separately. Thus, the sum of the scores is calculated to obtain an absolute value corresponding to SoC, ranging from 13 to 65 points.

QoL was assessed with the application of the PedsQL 4.0 Inventory^TM^. It consists of a set of generic questionnaires originally developed in English by Varni et al.^12^ and validated in Portuguese by Klatchoian et al.^13^ The questionnaire consists of 23 questions divided into four dimensions, namely, physical, emotional, social, and academic. The psychosocial dimension consists of the sum of the emotional, social, and school dimensions. Each item contains five response options on a Likert scale, whose values are later operationalized on a scale of 0–100, in which a higher score represents the best state.

Finally, the Economic Classification questionnaire of Brazil/2011 (ABEP) presents a range of socioeconomic classifications by assigning weights to a set of items of domestic comfort, also taking into account the level of education of the head of the household.

The data were processed and analyzed through the Statistical Package for Social Sciences (SPSS) version 21.0. The continuous variables are characterized in terms of their respective medians or means and standard deviations, while the categorical variables are described in absolute frequencies. To compare the SoC scores, as well as the total and per domain PedsQL between this sample and its variables of interest, the Student’s *t*-test, Mann-Whitney U test, F-test (ANOVA), and the Kruskal-Wallis test were carried out for independent samples. The Spearman’s correlation was also employed to evaluate the association between the QoL and SoC dimensions. Finally, a multiple linear regression was applied to verify the degree of influence of the SoC score in relation to the dimensions of QoL.

The reliability of the SoC and QoL questionnaires was assessed by internal consistency taking into account Cronbach’s alpha. The present study adopted a significance level of 5%, with a confidence interval of 95%.

The project was approved by the Ethics Committee in Research of the Institute of Integrated Medicine professor Fernando Figueira (IMIP), protocol no. 2830-12. All regulatory and formal procedures were followed, namely, the signature of the informed consent form (ICF) by the participants’ parents and/or guardians. Moreover, the present research was carried out in accordance with the rules of Resolution 466/12 of the National Council on Health Research involving human beings.^15^

## RESULTS

Regarding the sociodemographic data, among the 164 adolescents surveyed, 85 (51.8%) participants aged between 12 and 14 years, followed by 49 (29.9%) participants aged between 10 and 11 years, and 83 (50.6%) were male participants. The majority of the participants (84.1%) had not completed basic education. In turn, concerning the monthly household income (in minimum wages), 130 (79.3%) families earned between one and two times the minimum wage, while according to the Brazilian economic classification ABEP, 116 (70.7%) families were classified as Class C.

In relation to the distribution of heart diseases, heart valve diseases totaled 72.3% of the sample and 38.4% had a diagnosis of congenital heart disease. Moreover, 76 (46.4%) of those with valvopathy were associated with rheumatic fever (RF). The most frequent congenital anomalies were tetralogy of Fallot (20.7%), interventricular communication (15.2), interatrial communication (11.0%), and aortic coarctation (1.2%). The most common symptoms in adolescents were dyspnea (51.2%) and tachycardia (50.0%), followed by precordial pain (24.4%). It is also worth pointing out that heart diseases and the symptoms presented herein overlap. Therefore, many teenagers had more than one disease and associated symptoms.

Table 1 presents a significant difference (p<0.05) observed in the association between SoC and the clinical variables, when correlating symptoms such as dyspnea and tachycardia. Nevertheless, no statistically significant difference was observed between SoC and the sociodemographic variables considered (Table 1).

**Table 1.** Relationship between the Sense of Coherence and sociodemographic and clinical variables.

	Average±DP	p-value
Age group (years)		0.150^a^
9–11	51.31±6.60	
12–14	49.20±6.04	
15–18	50.60±5.99	
Gender		0.338^2^
Male	50.65±5.50	
Female	49.51±6.90	
Schooling		0.320^b^
Incomplete secondary education	49.88±6.30	
Complete secondary education/high school	51.17±6.18	
Social class (ABEP)		0.062^c^
B	53.75±4.18	
C	49.90±6.47	
D+E	49.47±5.75	
Income (MW)		0.493^c^
<1	50.94±6.37	
1–3	50.21±6.14	
≥3	48.44±6.88	
Diseases		
Valvopathy		0.463^b^
Yes	50.25±6.25	
No	49.64±6.26	
Congenital cardiopathy		0.156^b^
Yes	49.35±6.23	
No	50.54±6.23	
Symptoms		
Dyspnea		0.010^b^
Yes	48.98±6.17	
No	51.25±6.13	
Precordial pain		0.286^b^
Yes	49.30±5.95	
No	50.34±6.33	
Tachycardia		0.019^b^
Yes	49.01±5.98	
No	51.16±6.35	
Tachypnea		0.573^b^
Yes	48.80±6.60	
No	50.21±6.21	

^a^ F test (ANOVA); ^b^Mann-Whitney U Test; ^c^Kruskal-Wallis test.

The physical dimension was the most affected within QoL, showing an association with age, gender, and social class. Social class showed a correlation with all dimensions, except for the emotional dimension, which was not associated with any variable. In turn, the social, academic, and psychosocial dimensions showed an association with schooling (Table 2). Moreover, the symptoms referred by adolescents as dyspnea, tachycardia, and tachypnea were negatively associated with their QoL, since those who have these symptoms have a lower QoL (Table 3).

**Table 2.** Relationship between the dimensions of Quality of Life and sociodemographic data of adolescents.

	Dimensions of Quality of Life					
	Physical	Emotional	Social	Academic	Psychosocial	Total score
	Average±DP	Average±DP	Average±DP	Average±DP	Average±DP	Average±DP
Age group (years)						
9–11	69.83±18.81	74.18±19.16	77.45±19.58	63.27±15.90	71.63±16.09	71.01±16.30
12–14	72.17±18.65	75.53±17.39	76.88±20.22	63.00±16.24	71.80±14.76	71.93±15.20
15–18	81.25±14.36	74.17±16.25	85.17±14.88	68.50±16.56	75.94±11.73	77.79±10.74
p-value	**0.023^a^**	**0.875^a^**	**0.166^a^**	**0.258^b^**	**0.434^a^**	**0.165^a^**
Gender						
Male	76.43±18.04	76.39±16.84	80.54±18.79	63.49±16.15	73.47±14.48	74.50±14.83
Female	69.75±18.11	73.33±18.41	76.54±19.74	64.69±16.40	71.52±14.92	70.91±14.96
p-value	**0.018^c^**	**0.251^c^**	**0.179^c^**	**0.638^d^**	**0.412^a^**	**0.137^b^**
Schooling						
<12 years	71.99±18.55	74.71±18.03	76.67±19.45	62.39±15.92	71.26±15.02	71.51±15.27
≥12 years	79.69±16.64	74.79±15.57	88.96±15.25	73.33±15.51	79.03±11.01	79.26±11.84
p-value	**0.050^c^**	**0.922^c^**	**0.004^c^**	**0.007^c^**	**0.022^a^**	**0.024^b^**
Social class*						
B	83.07±18.24 ^(A)^	77.92±17.51	88.33±15.72 ^(A)^	76.67±19.46 ^(A)^	80.97±13.42	81.70±14.59
C	73.52±18.24 ^(A)^	75.86±18.28	80.09±18.81 ^(A)^	64.87±15.40 ^(A)^	73.61±14.36	73.58±14.69
D+E	68.58±17.65 ^(B)^	70.69±15.22	70.42±19.69 ^(B)^	57.36±15.05 ^(B)^	66.16±14.15	67.00±14.22
p-value	**0.041^c^**	**0.190^c^**	**0.007^c^**	**0.002^c^**	**0.003^b^**	**0.005^b^**
Income**						
<1	74.22±22.46	75.63±19.48	71.25±25.98	63.13±18.70	70.00±20.09	71.47±20.62
1–3	73.27±18.30	75.00±17.42	78.85±18.52	64.15±16.13	72.67±14.24	72.88±14.58
≥3	71.18±15.10	73.33±18.55	83.06±17.50	64.44±15.61	73.61±12.90	72.77±12.46
p-value	**0.779^c^**	**0.953^c^**	**0.385^c^**	**0.979^c^**	**0.903^b^**	**0.989^b^**

^a^ Kruskal-Wallis test; ^b^F test (ANOVA); ^c^Mann-Whitney U test; ^d^Student’s t test with equal variance. *ABEP classification; **the number of minimum wages. ***different superscript letters denote significant difference between corresponding categories.

**Table 3.** Relationship between the dimensions of Quality of Life and clinical data of the adolescent.

	Dimensions of Quality of Life					
	Physical	Emotional	Social	Academic	Psychosocial	Total score
	Average±DP	Average±DP	Average±DP	Average±DP	Average±DP	Average±DP
Diseases						
Valvopathy						
Yes	74.48±17.57	74.83±17.26	78.25±19.77	64.04±16.33	72.38±14.50	73.11±14.55
No	69.46±20.00	75.00±18.86	79.43±18.18	64.20±16.17	72.88±15.36	71.69±16.14
p-value	**0.167^a^**	**0.874^a^**	**0.817^a^**	**0.985^a^**	**0.869^a^**	**0.583^a^**
Congenital cardiopathy						
Yes	69.59±19.68	74.21±18.01	79.92±18.68	62.86±16.13	72.33±14.97	71.38±15.88
No	75.34±17.17	75.30±17.49	77.72±19.74	64.85±16.33	72.62±14.58	73.57±14.37
p-value	**0.067^a^**	**0.668^a^**	**0.479^a^**	**0.446^b^**	**0.892^a^**	**0.363^a^**
Symptoms						
Dyspnea						
Yes	66.29±17.09	71.49±18.35	74.88±19.34	61.37±15.74	69.25±14.49	68.22±14.52
No	80.31±16.86	78.44±16.24	82.44±18.62	66.94±16.35	75.94±14.18	77.46±13.99
p-value	<0.001^a,^*	0.018^a,^*	0.010^a,^*	**0.054^a^**	0.004^a,^*	<0.001^a,^*
Precordial pain						
Yes	68.20 ±16.03	71.75±15.26	80.50±16.04	63.13±14.31	71.79±12.30	70.54±12.07
No	74.72±18.80	75.89±18.29	77.94±20.27	64.40±16.85	72.74±15.42	73.43±15.76
p-value	0.040^a,^*	**0.155^a^**	**0.589^a^**	**0.694^a^**	**0.543^a^**	**0.212^a^**
Tachycardia						
Yes	65.13±15.65	72.07±16.70	72.99±19.15	58.90±14.70	67.99±13.39	66.99±13.03
No	81.14±17.36	77.68±18.21	84.15±17.91	69.27±16.12	77.03±14.61	78.46±14.62
p-value	<0.001^a,^*	0.035^a,^*	<0.001^a,^*	<0.001^b,^*	<0.001^a,^*	<0.001^a,^*
Tachypnea						
Yes	68.75±12.61	74.67±16.20	68.33±18.77	51.00±8.49	64.67±10.22	66.09±9.36
No	73.57±18.78	74.90±17.83	79.60±19.12	65.40±16.26	73.30±14.86	73.40±15.27
p-value	**0.196^a^**	**0.881^a^**	0.023^a,^*	<0.001^a,^*	0.021^a,^*	0.040^a,^*

^a^ Mann-Whitney U test; ^b^Student’s *t*-test with equal variances. *Significant difference at the 5.0% level.

From these results, it can be inferred that all dimensions are positively correlated with the SoC (p<0.001), according to the analysis of the Spearman’s correlation coefficient. Regarding the multiple linear regression analyses, the impact of the SoC seems especially high for the educational and emotional dimensions (<0.001), followed by the social dimension (p=0.02) (Table 4). It was not possible to insert the psychosocial dimension in the model, since it is a sum of the emotional, academic, and social dimensions.

**Table 4.** Correlation and impact power of the Sense of Coherence and the areas of Quality of Life.

Dimension	Spearman correlation	Multiple linear regression
	s (p-value)	
Physical	0.484 (<0.001)	-0.052 (0.111)
Emotional	0.515 (<0.001)	0.117 (<0.001)
Social	0.482 (<0.001)	0.066 (0.025)
Academic	0.505 (<0.001)	0.131 (<0.001*)
Psychosocial	0.603 (<0.001)	–
Total score	0.550 (<0.001)	

*Statistically different from zero and significant to 5.0%.

## DISCUSSION

Still the use of the SoC questionnaire in adolescents is recent. The first study that discussed this issue was carried out by Freire et al.^16^, having studied oral health and its association with the SoC in adolescents. Thus, it was not possible to compare the results obtained in this study with other results involving young Brazilians with heart disease.

Nevertheless, the results are consistent with recent studies found in the literature for other countries. Neuner et al.^7^ used the same instrument in German, with an SOC-L9 consisting of nine questions in a 1–7 Likert scale, with an average SoC of 48.9 and a possible range of 9–63. In turn, Apers et al.^8^ used a shortened and adapted version of the SoC-13, which was measured on a 1–7 Likert scale, ranging from 13 to 91, resulting in an average SoC of 53.6. Moons et al.^17^ carried out a study with 4,028 adults with congenital heart disease from different countries, assessed the SoC using the 13-item Life Orientation Questionnaire, also known as the 13-SOC scale, with a score ranging from 13 to 91, showing an average SoC of 65.5 – higher than the result found in the present sample. Taking into account the average SoC and the respective intervals previously mentioned, the result for the sample studied in the present work is considered high (average SoC of 50.09 and range 35–62).

Several studies revealed that the SOC in a group of adolescents with heart disease was higher than in the group of healthy adolescents.^6–8^ Growing up with a chronic illness can have a positive influence on the development of the SOC, which allows a better adaptation and the creation of strategies for dealing with the condition.^18^

In this research, when analyzing the differences between the measurements of SoC according to gender, no statistically significant difference was observed (p=0.338). However, the SoC of girls was slightly lower than of boys. In previous research studies^6–8^ conducted with adolescents with heart disease, significant differences were found for SoC between boys and girls, with the latter group showing significantly lower scores. In general, girls experience higher levels of interpersonal stress and are more sensitive than boys, tending to exhibit more negative emotional responses, such as anxiety and depression, which may reduce the SoC in girls.^19^

Regarding the presence of symptoms, the findings of Wang^6^ corroborate the results presented herein, in which the symptoms resulting from heart diseases, such as dyspnea and tachycardia, were significantly associated to the SoC (p<0.05), since adolescents with such symptoms exhibit a lower SoC. This result suggests that SoC acts as a protective factor. Thus, if the SoC is influenced by these symptoms, the restrictions resulting from the disease do not seem to interfere with the ability of dealing with physical limitations.

The cardiac pathologies studied herein were not significantly associated with SoC. Valvular heart disease (73.2%) presented a higher prevalence when compared to congenital heart disease (38.4%), being mainly associated to RF.

According to Rothenbühler et al.,^20^ RF is the leading cause of acquired heart disease in children and young adults in developing countries, whose more relevant sequelae is rheumatic valve disease. Rheumatic valve disease is a pathology often associated with lower socioeconomic conditions, such as the population studied in the present study. Moreover, it is also related to poor housing and inadequate medical care, most frequently affecting patients aged between 5 and 15 years. Data obtained from the Informatics Department of the Single Brazilian Health System show that in Brazil, despite the lack of reliable statistical data, RF is present in 0.3–3.0% of the susceptible population. One-third of these cases leads to chronic lesions of the cardiac valves, corresponding to approximately 6,000 new cases of chronic heart disease a year.^21^

Most studies analyzed showed a better QoL of adolescents with heart disease when compared to a healthy group.^7,8,20^ These results may reflect in an often adaptation to the situation by the part of these young people, reinforcing the hypothesis that the QoL of this population is not proportional to the severity of the disease. Moreover, Reiner et al.^22^ also highlighted that these individuals require a significant amount of additional care, which may promote a protective and cohesive environment, reducing stress, and promoting positive adaptation.

Among the dimensions presented in the PedsQL, the school dimension showed the lowest score. Due to long periods of hospitalization, the need of going back and forth to doctors for reassessments, as well as the possible recurrence of pain and fatigue caused by the surgery in itself, adolescents often found themselves oppressed by the loss of school classes. Therefore, the damage is considered almost inevitable.

A study conducted by Terreri et al.^23^ in São Paulo, Brazil, with 100 patients aged up to 18 years old diagnosed with RF revealed that, since the beginning of the disease, 84 patients had lost 1,812 days of classes at school (an average of 21.6 days/patient). The absence of these students was mainly due to the inability of going to school due to health problems, including medical appointments and examinations. Therefore, the damage at school leads to single and social losses, affecting their current and future QoL.^24^

Regarding the type of heart disease and QoL, the present findings are in accordance with several studies that showed no relationship between this variable and the QoL.^7,25^

QoL was significantly affected by symptoms such as dyspnea, tachycardia, and tachypnea (p<0.05). The symptoms associated to cardiovascular disease may lead to limiting physical, psychological, and social sequelae. These sequelae may restrict the daily routine of adolescents, as well as their social life, as they often cannot take part in the same physical activities as their friends from the same age. In this scenario of limitation, adolescents victimized by the disease, beyond a physical aspect, experience psychological conflicts, which also affect their emotional socialization.^26^

The physical dimension of QoL was associated with age, gender, and social class, variables that do not normally alter the adolescents’ physical capacity. However, it is worth pointing out that the functional indices, such as capacity for cardiopulmonary exercise or exercise tolerance are not sufficient to reflect the QoL regarding individual subjective perception.^27^ Contrary to the health status that often reflects problems with limitations of functioning, QoL includes dimensions that involve the physical, the psychological, and the social,^28^ which can be influenced by age, sex, and the social class that the individual is inserted.^27^

Similar to the recent studies, a positive correlation (p<0.001) was observed between SoC and all dimensions of QoL, which makes these adolescents more resilient and able to find a solution to deal with their condition. Thus, the SoC is a protective factor against the adversities of daily chronic stress, thereby promoting a better QoL.^7,9^

Eriksson and Lindstrom,^5^ through a systematic review of 32 publications assessing the relationship between SoC and QoL in various samples, such as patients with human immunodeficiency virus, cancer, and heart and respiratory diseases, among other diseases, found that the stronger the SoC, the higher the QoL. A high correlation (r>0.50) was observed in all studies, when applying specific measurement instruments, while slightly lower in studies using more generic instruments. Thus, despite the use of a generic instrument in the present study, a good correlation of between 0.48 and 0.60 was found. The results from longitudinal studies also corroborated the findings of transversal studies, with the salutogenic theory considered a factor for promoting QoL.^29^

Despite the significant association between SoC and all dimensions assessed (p<0.001), the physical dimension showed a low correlation with the variables analyzed. This finding can be confirmed by the fact that symptoms, such as dyspnea and tachycardia, framed within the physical dimension, are associated with lower QoL and SoC scores. Thus, the functional order constraints resulting from heart disease jeopardized the physical well-being of adolescents, as well as their ability to adapt to this situation.^20^

Confirming the results from Spearman’s correlation and adjusting potential confusion factors, the multiple linear regression revealed that the SoC score did not influence the physical dimension as in the assessment of QoL. Similar results were found by Eriksson and Linsdström^14^ in the association between SoC scores and health revealing a stronger influence of the SoC score on mental than physical health. Thus, this indicates that the SoC score seems to be a tool for health promotion and the development of a subjective state of health.^3,17^ These results point to the need for developing interventions that provide a positive impact on these adolescents, tackling their functional limitations.

When applying the linear regression model, the SoC had a greater impact on the educational and emotional dimensions. During adolescence, school plays a central role in the everyday life of teenagers. In this regard, considering that the school dimension had lower scores in the perception of QoL in the bivariate analysis, the SoC can be a factor that can be potentially worked in this population, aimed at training individuals to deal with possible barriers, leading to a successful process of adaptation.

Although it is possible to identify the most affected dimensions in the perception of QoL as well as in the context in which the SoC has a greater influence, cardiac adolescents cannot be analyzed separately. The QoL covers an interconnected multidimensional concept, thus requiring a comprehensive and integral evaluation.

The findings of the present study corroborate the salutogenic theory, which considers that a greater ability to cope with the difficulties of life leads to more favorable health consequences, as well as a stronger SoC and a better quality in the individual’s life.^3^ Some studies reported that a high SoC score is one of the factors responsible for a good QoL in patients with heart disease, despite all the limitations presented by them.^29^ Thus, improving the SoC through interventions and prevention measures may potentially improve the QoL and health perception among adolescents with chronic diseases, namely heart disease.^30^

The main limitation of this research lies on the investigation design, since it is not possible to establish cause-and-effect relationships with transversal studies. As the research was carried out in a referral center for adolescents with heart disease, it was not possible to form a comparison group with healthy adolescents, which was another limitation of the present study.

This study demonstrates that the SoC is a protective factor for adolescents. This factor helps to improve the perception of QoL and to successfully deal with daily adversities and chronic stress.
